# Intergenerational transmission of alloparental behavior and oxytocin and vasopressin receptor distribution in the prairie vole

**DOI:** 10.3389/fnbeh.2015.00191

**Published:** 2015-07-23

**Authors:** Allison M. Perkeybile, Nathanial Delaney-Busch, Sarah Hartman, Kevin J. Grimm, Karen L. Bales

**Affiliations:** ^1^Department of Psychology, University of California, DavisDavis, CA, USA; ^2^The Kinsey Institute for Research in Sex, Gender and Reproduction, Indiana UniversityBloomington, IN, USA; ^3^Department of Psychology, Tufts UniversityMedford, MA, USA; ^4^Department of Human Development, University of California, DavisDavis, CA, USA; ^5^Department of Psychology, Arizona State UniversityTempe, AZ, USA

**Keywords:** alloparental behavior, parental care, prairie vole, oxytocin receptor, vasopressin receptor, intergenerational transmission, natural variation

## Abstract

Variation in the early environment has the potential to permanently alter offspring behavior and development. We have previously shown that naturally occurring variation in biparental care of offspring in the prairie vole is related to differences in social behavior of the offspring. It was not, however, clear whether the behavioral differences seen between offspring receiving high compared to low amounts of parental care were the result of different care experiences or were due to shared genetics with their high-contact or low-contact parents. Here we use cross-fostering methods to determine the mode of transmission of alloparental behavior and oxytocin receptor (OTR) and vasopressin V1a receptor (V1aR) binding from parent to offspring. Offspring were cross-fostered or in-fostered on postnatal day 1 and parental care received was quantified in the first week postpartum. At weaning, offspring underwent an alloparental care test and brains were then collected from all parents and offspring to examine OTR and V1aR binding. Results indicate that alloparental behavior of offspring was predicted by the parental behavior of their rearing parents. Receptor binding for both OTR and V1aR tended to be predicted by the genetic mothers for female offspring and by the genetic fathers for male offspring. These findings suggest a different, sex-dependent, role of early experience and genetics in shaping behavior compared to receptor distribution and support the notion of sex-dependent outcomes.

## Introduction

Alterations in the early life environment of a developing animal can have long-term consequences on behavior. Manipulations of offspring in early life, such as repeated brief handling and long-term separation, have long been known to alter various adult behaviors and hypothalamic-pituitary-adrenal (HPA) axis response to stressors (Levine, 1957; Denenberg et al., 1962; Levine et al., 1967; Plotsky and Meaney, 1993; Ladd et al., 1996; Boccia and Pedersen, 2001; Padoin et al., 2001) in the rat. Varied early handling in the prairie vole in the first week of life also has consequences on offspring behavior, including sex-dependent changes in alloparental care and partner preference formation (Bales et al., 2007a) and later parental behavior (Stone and Bales, 2010).

Even without experimental manipulation of the early environment, natural variations in early experience can have long-term consequences; these natural variations include differences in the type of care as well as differences in the number of caregivers present. For example, rat dams display varying levels of maternal care of offspring. Offspring reared by high licking and grooming (LG) dams display decreased reactivity to novel environments (Liu et al., 1997; Caldji et al., 1998; Francis et al., 1999) and females that receive high LG behavior (compared to those that receive low LG) later display high LG behavior toward their own offspring (Francis et al., 1999). Cross-fostering studies in rats indicate that adult outcomes are transmitted in a non-genomic fashion and that it is the early care that drives later maternal behavior in female offspring of high and low LG dams (Francis et al., 1999; Champagne et al., 2003). Impaired care in mice results in altered behavior in female offspring and that care style is also transmitted in a non-genomic fashion (Curley et al., 2008), similar to the rat model.

Many of these same early life changes also have lasting effects on neuropeptide systems, particularly oxytocin (OT) and vasopressin (AVP), two peptides that are involved in various social and parental behaviors. Following rearing by high LG dams in rats, adult offspring show sex-dependent increases in oxytocin receptor (OTR) and AVP V1a receptor (V1aR) binding in regions known to be involved in the display of parental behavior (Francis et al., 2002). Differences in binding patterns are also observed with varying the number of animals providing early-life care, such as biparental rearing in the prairie vole (Ahern and Young, 2009) and communal rearing in mice (Curley et al., 2009a; Branchi et al., 2013), as well as with delayed weaning of offspring (Curley et al., 2009b). Early experimental manipulation of offspring has consequences for these systems as well. Daily 3-h maternal separation alters both OTR and V1aR binding in various regions in male rats (Lukas et al., 2010), while reduced early handling results in increased OTR binding in female prairie voles (Bales et al., 2011).

Taken together, it appears that relatively small or brief changes in the early environment, including both experimental manipulations and naturally occurring variation, have lasting impacts on the organization of OT and AVP systems. These systems are heavily implicated in the displays of various social behaviors. While there is evidence in the rat that OTR and V1aR binding patterns are transmitted from one generation to the next, there is little evidence of paternal transmission of receptor binding, a result that is, perhaps, not surprising given the limited role of the father in most mammalian species. This study aims to investigate the varying roles of maternal and paternal influences on offspring behavior and neurobiology in the prairie vole, and to determine if these influences differ between male and female offspring.

The prairie vole is a small rodent native to the Midwestern United States that displays social monogamy, with the father heavily involved in care of the offspring (Kleiman, 1977). In addition, the organization and function of the OT and AVP systems have been well characterized in this species (Insel and Shapiro, 1992a; Insel et al., 1994; Wang et al., 1996), including their relation to alloparenting (Olazábal and Young, 2006a, b; Keebaugh and Young, 2011) and developmental experiences (Carter, 2003; Bales and Perkeybile, 2012). Prairie voles are sensitive to both behavioral and pharmacological early life manipulations, including changes in social behaviors (Stribley and Carter, 1999; Bales and Carter, 2003a, b; Cushing et al., 2005; Bales et al., 2007a) and neuropeptide systems (Yamamoto et al., 2004; Kramer et al., 2006). In particular, changes in receptor binding have been observed following early manipulations, including sex-dependent differences after an acute early-life OT exposure (Bales et al., 2007b) and early handling (Bales et al., 2011). The presence of the father in the nest and the active role fathers often take in caring for offspring provides an opportunity to investigate the role of early paternal care in shaping both offspring behaviors and neuropeptide systems.

We have previously demonstrated reliable variation in biparental care directed toward offspring in the first postnatal days (Perkeybile et al., 2013). High- and low-contact breeder pairs differ in both the amount and the type of care given to offspring, and these differences in early life care correlate with differences in social behavior in the adolescent offspring. It is unclear, however, whether the differences seen in later social behavior in the offspring are a result of differing early experiences between high- and low-contact offspring or rather are a consequence of shared genetics with the parents—high-contact parents may potentially pass on traits to offspring through genomic mechanisms that promote increased amounts of social behavior. Previous cross-fostering work in several vole species indicates that they are sensitive to changes brought about by interspecies rearing experiences. For instance, meadow vole offspring reared by prairie vole parents showed an adult social preference for prairie rather than meadow voles (McGuire and Novak, 1987) while prairie vole offspring cross-fostered to montane vole parents did not survive to weaning (Shapiro et al., 1989).

Using within-species cross-fostering methods, in this study we examined the relationship between early parental care and post-weaning alloparenting behavior in offspring, as well as the OTR and V1aR systems in parents and offspring. Upon weaning, we measured alloparental care in the offspring to determine if the early care environment predicted later alloparenting behaviors. Alloparental behavior is seen in non-reproductive male and female prairie voles in the wild and has consequences for the breeding pair, the pups, and the alloparent themselves. Field studies have found that offspring of breeding pairs may remain in the natal nest following weaning (Getz and Mcguire, 1997) and will participate in the care of subsequent litters born, engaging in parental-like behaviors such as licking and grooming, huddling over pups, and pup retrieval (Solomon, 1991; Wang and Novak, 1992; Roberts et al., 1998a). This care by the alloparent is linked to shorter interbirth intervals for the breeding pair (Solomon, 1991) and results in faster development of the pups (Solomon, 1991, 1994). In addition, alloparental experience prior to reproduction can facilitate later parental care of their own offspring (Roberts et al., 1998b; Stone et al., 2010). Alloparental behavior is not simply a response to a novel stimulus in the environment; male prairie voles presented with a small wooden dowel investigated the dowel but did not display the full range of parental-like behavior as they did when exposed to a pup (Kenkel et al., 2012). There is evidence that early handling manipulations alter alloparental behavior in male prairie voles (Bales et al., 2007a) and we have previously shown that variation in early parental care is related to altered alloparenting in young male offspring (Perkeybile et al., 2013). Therefore, we predicted that male offspring would be particularly sensitive to changes in the early environment and would be more likely to resemble the rearing style of their foster rather than birth parents compared to the female offspring.

Receptor binding patterns for OTR and V1aR were also examined in both the parents and offspring. There is evidence in rats that binding patterns are transmitted to offspring via non-genomic mechanisms and that this transmission is sex-specific for each neuropeptide (Francis et al., 2000, 2002). In addition, female prairie voles showed changes in OTR binding in an early handling paradigm (Bales et al., 2011), indicating that short-term early manipulations can have lasting impacts. Therefore, we expected that females would have OTR binding patterns and males would have V1aR binding patterns reflective of their rearing parents and that changes in males would be more pronounced than those in females, indicating that neuropeptide receptor binding patterns are a product of experience rather than genetics.

## Materials and Methods

### Subjects

Subjects were laboratory-bred prairie voles (Microtus ochrogaster), descendants of a stock originally wild-caught near Champaign, IL, USA. Breeder pairs were housed in large polycarbonate cages (44 cm × 22 cm × 16 cm). Food (high-fiber Purina rabbit chow) and water were available ad libitum and cotton squares were provided for nesting material. Upon weaning on postnatal day (PND) 20, weanlings were housed in same-sex pairs in small polycarbonate cages (27 cm × 16 cm × 16 cm). Animals were maintained on a 14:10 light:dark cycle. All procedures were reviewed and approved by the Institutional Animal Care and Use Committee of the University of California, Davis, CA, USA.

### Cross-Fostering and Parental Care Observations

Within 24 h of birth, infants were briefly removed from the nest and were weighed, dye marked for individual identification with Nyanzol dye, and sexes were checked. If necessary, litters were culled to four pups. Infants were then either returned to their natal nest or cross-fostered to an unrelated breeder pair. Each breeder pair was then rearing two of their own in-fostered offspring and two unrelated cross-fostered offspring (one male and one female in-fostered, one male and one female cross-fostered, when possible). In total, 10 breeder pairs reared 18 in-fostered offspring (9 males; 9 females) and 20 cross-fostered offspring (11 males; 9 females).

Focal observations for each pup were conducted to characterize the amount and type of parental care given to each offspring. Ten minute observations occurred over 3 days in the first week postpartum (observation 1: PND 1–2; observation 2: PND 3–4; observation 3: PND 6–7), with each day including a morning and an afternoon observation. Animals remained undisturbed in the home cage during all observations. Observations were conducted live by two trained observers using Behavior Tracker 1.5^1^ and included six pup-directed parental behaviors observed in both the mother and the father as well as three additional maternal nursing postures using a previously established ethogram (Stone and Bales, 2010; see Table 1). Males were distinguished from females within breeding pairs by features such as size, individual color and markings, or the presence of offspring attached to the nipple. This observation paradigm has previously been used by our laboratory to quantify parental behavior in the prairie vole (Perkeybile et al., 2013). Offspring were weighed again on PND 7 following the last behavioral observation.

**Table 1:** **Parental behavior ethogram used during characterization of early parental care, based on Stone and Bales (2010)**.

Parental behaviors (maternal and paternal)	Description
Huddling	All four paws touching ground; holding self up over pups; head tucked, back arched
Non-huddling contact	In contact with pups and quiescent
Hunch	Sitting up on hind limbs in a hunched position; forelimbs off the ground; pups in front
Licking/Grooming	Licking and grooming pups
Anogenital Licking/Grooming	Licking and grooming pups, specifically the anogenital region
Retrieval	Lifting pup in mouth and moving it at least one inch
**Maternal postures**	**Description**
Active nursing	Pups attached while locomoting around home cage
Lateral nursing	Laying on side with pups laying in front
Prone nursing	Standing over pups in a relaxed position without locomotion

### Alloparental Care

Between PND 23–25 male and female subjects were tested with a novel pup to measure alloparental care behavior (Roberts et al., 1996). The testing arena consisted of two small polycarbonate cages (27 cm × 16 cm × 16 cm) connected by a short clear tube. Subjects were placed in the arena for a 45 min acclimation period. After this time, a novel pup (PND 0–4) was placed in one cage. During the 10 min test, the subject was free to spend time interacting with the pup or alone in the empty cage. The tests were video-recorded and later scored by a trained observer using Behavior Tracker. Behaviors scored included time spent in the front cage, time spent in the back cage, time spent in the connecting tube, latency to approach the pup, time spent huddling over the pup, time spent in a pseudohuddling posture, time in non-huddling contact with the pup, time spent sniffing the pup, time spent licking/grooming the pup, autogrooming, the number of pup retrievals, and the latency to attack the pup. Attacks were rare. When they occurred, the test was immediately stopped and the subject was removed from the arena. Pups were checked for any injuries. If possible, injuries were treated and the pup was returned to their home cage. If necessary, the pup was euthanized. Pups were used for no more than two test sessions and were then returned to their home cage.

### Receptor Autoradiography

Following alloparental care testing (offspring PND 24–26), all parents and offspring were deeply anesthetized using isoflurane gas and were then euthanized by cervical dislocation and rapid decapitation. Brains were removed, flash-frozen and stored at −80°C. Brains were sectioned at 20 μm into six series, mounted onto Super-frost slides and stored at −80°C until assayed. Slides were allowed to thaw at room temperature and were then fixed in 0.1% paraformaldehyde (7.4 pH) for 2 min. Slides were then washed twice for 10 min in 50 mM Tris-HCl buffer solution (7.4 pH), followed by incubation in the tracer buffer at room temperature for 60 min. Tracer buffer consisted of 50 mM Tris-HCl buffer (7.4 pH) with 10 mM MgCl_2_, 0.1% bovine serum albumin, and 50 pM of radiotracer. For OTR binding [^125^I]-ornithine vasotocin analog [(^125^I)OVTA] [vasotocin, d(CH_2_)_5_[Tyr(Me)^2^, Thr^4^, Orn^8^, (^125^I)Tyr^9^-NH_2_]; 2200 Ci/mmol] was used (NEN Nuclear, Boston, MA, USA). For V1aR binding ^125^I-lin-vasopressin [^125^I-phe- nylacetyl-D-Tyr(ME)-Phe-Gln-Asn-Arg-Pro-Arg-Tyr-NH_2_] (NEN Nuclear) was used. Following the incubation period, slices were rinsed in 50 mM Tris-HCl buffer (7.4 pH) with 10 mM MgCl_2_ at 4°C four times for 5 min each, followed by a 30 min rinse in the same solution at room temperature while agitating on a shaker plate. Sections were briefly dipped in 4°C dH_2_O and were then rapidly dried with a stream of cool air. The following day slides were exposed to Kodak BioMaxMR film (Kodak, Rochester, NY, USA) with ^125^I microscale standards (American Radiolabeled Chemicals, Inc., St., Louis, MO, USA). Slides were exposed for 168 h for OTR binding and 96 h for V1aR binding. Receptor binding was quantified from film using NIH Image J. The ^125^I microscale standards were used to convert uncalibrated optical density to disintegrations per minute (DPM). Areas of interest were quantified on both sides of the section and sides were compared for any differences. A measure of non-specific binding (NSB) was also taken for each section. The NSB value was subtracted from the binding value for each section and a mean was then calculated for each section, followed by a mean for the entire area for each subject. Area means were used in data analysis.

### Data Analysis

Residuals were checked for normality and, when necessary, were transformed using a square root transformation. Significance levels were set at *p* < 0.05. All analyses were performed using SAS 9.2. Multivariate analysis of variance (MANOVA) was used to compare differences in early care between cross-fostered and in-fostered offspring and between male and female offspring to ensure similar care was directed toward all offspring. Weights on PND 7 were analyzed using a mixed model ANOVA with sex, foster status, and a sex by foster interaction. Breeder pair was included as a random variable.

To reduce the number of behavioral variables, behaviors observed during early parental care observations were grouped to provide composite scores for each parent, resulting in variables including active maternal care, passive maternal care, non-huddling maternal nursing, active paternal care, and passive paternal care. The same was done with behavior in the alloparental care test, resulting in active and passive alloparental behavior scores. These composite scores, listed in Table 2, were used for correlation and regression analyses as well as analysis of mode of behavioral transmission. A Pearson correlation was used to examine the relationship between early parental behavior received and OTR and V1aR binding patterns in offspring in regions known to be involved in parental and alloparental behavior, including the central nucleus of the amygdala (CeAmy), bed nucleus of the stria terminalis (BNST), medial preoptic area (MPOA), lateral septum (LS), nucleus accumbens (NAcc) core and shell. A Pearson correlation was also used to look at the relationship between behavior in the alloparental care test and OTR and V1aR binding in these same regions. The relationship between care received in early life and later behavior in an alloparental care test were examined with a multiple regression. A false discovery rate correction was used for both correlation and regression analyses to correct *p*-values for multiple comparisons (Benjamini and Hochberg, 1995).

**Table 2:** **Composite scores of early parental behaviors and offspring alloparental behaviors**.

Parental behavior composites	Included behaviors
Active maternal care	Licking, anogenital licking, retrieval
Passive maternal care	Huddle, non-huddling contact
Non-huddling nursing	Prone nursing, active nursing, lateral nursing, hunched nursing
Active paternal care	Licking, anogenital licking, retrieval
Passive paternal care	Huddle, non-huddling contact, hunched posture
**Alloparental behavior composites**	**Included behaviors**
Active alloparental behavior	Sniffing, licking, retrieval
Passive alloparental behavior	Huddle, pseudohuddle, non-huddling contact

Transmission of OTR and V1aR density from parent to offspring was investigated to determine if binding patterns in offspring resembled those of biological parents or foster parents. Analyses were run separately for male and female offspring because the primary interest was in effects of rearing for each sex. Because half of all offspring were in-fostered, the assumption of independence between biological and foster parents needed for a multiple regression model was not attainable. Therefore, a cross-classified mixed model regression was used (Beretvas and Murphy, 2013). A fixed intercept model was used for data where there was very little covariance attributable to either the biological family or the rearing family, while a random intercept model was used when there was covariance attributable to these sources, with the dominant source of variance included as the nested factor. The same approach was used to analyze transmission of parental behavior from parent to offspring. For both analyses of receptor transmission and of behavior transmission, data points were normally distributed. Because of the large number of trend relationships present in these regression analyses, probabilities from each independent significance test were combined using Fisher’s combined probability test (Fisher, 1954) to test the overall significance for both the behavior and the receptor binding regression analyses. In addition, *post hoc* power analyses were conducted to explore whether trend-level findings were due to lack of power. For data analyzed with a fixed intercept model, multiple regression power analyses were conducted, while Monte Carlo simulations were done for data analyzed using a random intercept model. In all cases, results of the power analyses indicated powers at or above 0.80, suggesting that the tests were adequately powered.

## Results

**Figure 1:**
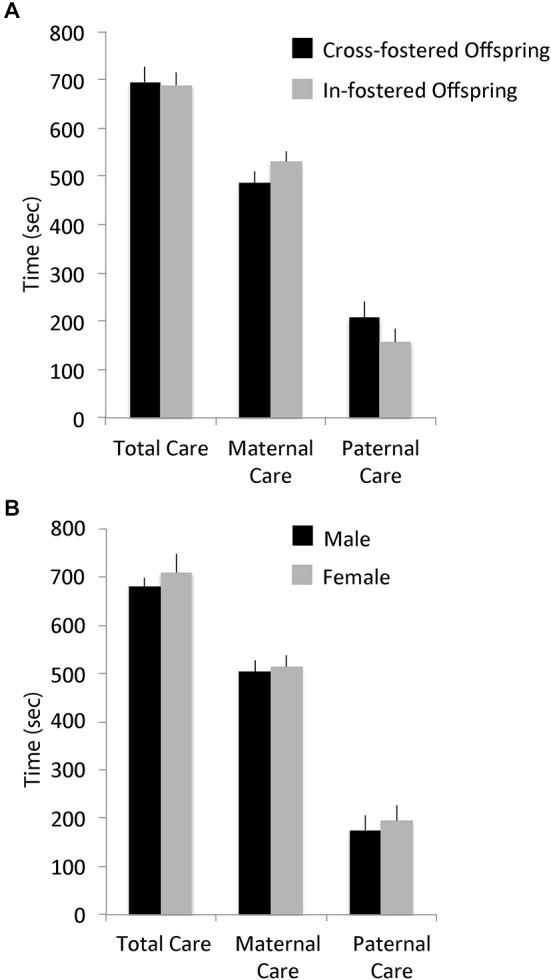
**Early parental care**. Early total care, maternal care, and paternal care of offspring did not differ by **(A)** foster condition or **(B)** offspring sex.

There were no differences in total pup-directed parental care in the first week postpartum between cross-fostered and in-fostered offspring or between male and female offspring (See Figure 1). MANOVA showed no significant effects of fostering condition (*F*_(14,22)_ = 1.53, *p* = 0.1769; total care, in-fostered: mean = 692.73, S.E. = 24.63, cross-fostered: mean = 698.87, S.E. = 31.89) or of offspring sex (*F*_(14,22)_ = 1.06, *p* = 0.4407; total care, male: mean = 1188.39, S.E. = 29.24, female: mean = 1226.56, S.E. = 54.24) on the type or amount of early parental care received. Offspring weights on PND 7 also did not differ by fostering condition (*F*_(1,36)_ = 0.05, *p* = 0.8199; in-fostered: mean = 6.01, S.E. = 0.17, cross-fostered: mean = 5.96, S.E. = 0.18) or by sex (*F*_(1,36)_ = 0.39, *p* = 0.5355; male: mean = 6.06, S.E. = 0.19, female: mean = 5.90, S.E. = 0.17).

**Figure 2:**
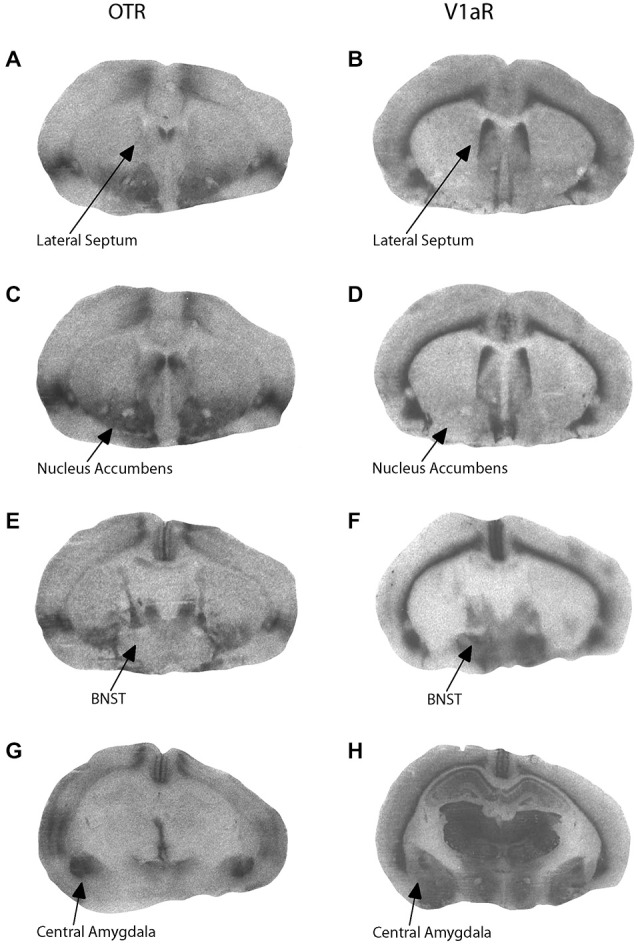
**Representative autoradiograms for oxytocin receptor (OTR) and vasopressin V1a receptor (V1aR) binding at the level of the lateral septum (LS; A,B), nucleus accumbens (C,D), bed nucleus of the stria terminalis (E,F), and central amygdala (G,H)**.

Components of early parental care received correlated with later neuropeptide receptor binding patterns in adolescent offspring. Representative OTR and V1aR autoradiographs from one female offspring are presented in Figure 2. Active maternal care in early life was negatively correlated with V1aR binding in the CeAmy in male offspring (*r* = −0.5889, adjusted *p* = 0.0404) and tended to be positively correlated with OTR binding in the BNST in female offspring (*r* = 0.5687, adjusted *p* = 0.0864). In addition, passive paternal care tended to be negatively correlated with V1aR binding in the BNST in male offspring (*r* = −0.5973, adjusted *p* = 0.0584). There were no significant correlations between behavior in the alloparental care test and OTR or V1aR binding in the CeAmy, BNST, MPOA, LS, or NAcc core or shell. Alloparental behaviors of offspring were predicted by early passive paternal care in a sex-specific manner. Increased early passive paternal care predicted increased sniffing of the infant during an alloparental care test by female offspring (*t*_(16)_ = 2.28, adjusted *p* = 0.0416) as well as more infant retrievals by male offspring (*t*_(18)_ = 2.38, adjusted *p* = 0.0320).

**Figure 3:**
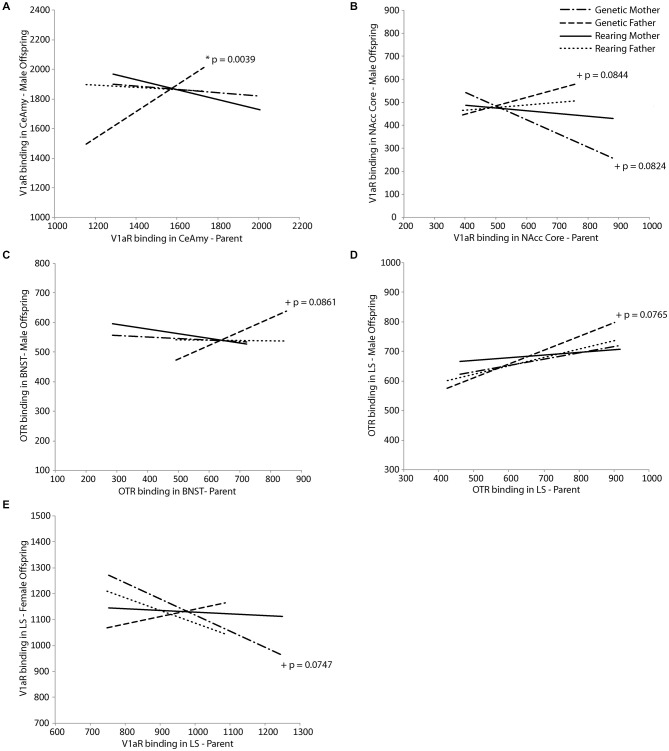
**Intergenerational transmission of neuropeptide receptor density**. V1aR density in male offspring is predicted by **(A)** V1aR density in the genetic father in the central nucleus of the amygdala (CeAmy; *t* = 3.50, *p* = 0.0039) and **(B)** tends to be predicted by V1aR density in the genetic mother (*t* = −2.57, *p* = 0.0824) and genetic father (*t* = 2.54, *p* = 0.0844) in the nucleus accumbens (NAcc) core. There are trends for OTR density in male offspring to be predicted by **(C)** OTR density in the genetic father in the bed nucleus of the stria terminalus (BNST; *t* = 1.93, *p* = 0.0861) and **(D)** in the lateral septum (LS; *t* = 2.03, *p* = 0.0765). V1aR density in female offspring tends to be predicted by **(E)** V1aR density in the genetic mother in the LS (*t* = −2.50, *p* = 0.0747).

Neuropeptide receptor binding patterns in the genetic but not rearing parents in regions involved in parental behavior were predictive of receptor binding patterns in offspring in a sex-dependent manner. For male offspring, V1aR density in the CeAmy was positively predicted by V1aR density of the genetic father in the same region (*t*_(18)_ = 3.50, *p* = 0.0039; Figure 3A), while V1aR density in the NAcc core tended to be positively predicted by V1aR density in the NAcc core of the father (*t*_(18)_ = 2.54, *p* = 0.0844) and negatively predicted by the genetic mother (*t*_(18)_ = −2.57, *p* = 0.0824; Figure 3B). In addition, OTR density in the BNST and LS of male offspring tended to both be positively predicted by OTR density in the genetic father in the BNST (*t*_(18)_ = 1.93, *p* = 0.0861; Figure 3C) and the LS (*t*_(18)_ = 2.03, *p* = 0.0765; Figure 3D), respectively. In female offspring, V1aR density in the LS tended to be negatively predicted by V1aR density of the genetic mother in the same region (*t*_(16)_ = −2.50, *p* = 0.0747; Figure 3E). Results of Fisher’s combined probability test indicate that the calculated *X*^2^ test statistic is greater than the expected test statistic (36.26426 < 35.94876), suggesting that receptor binding density in offspring is, in fact, predicted by that of their same-sex genetic parent.

**Figure 4:**
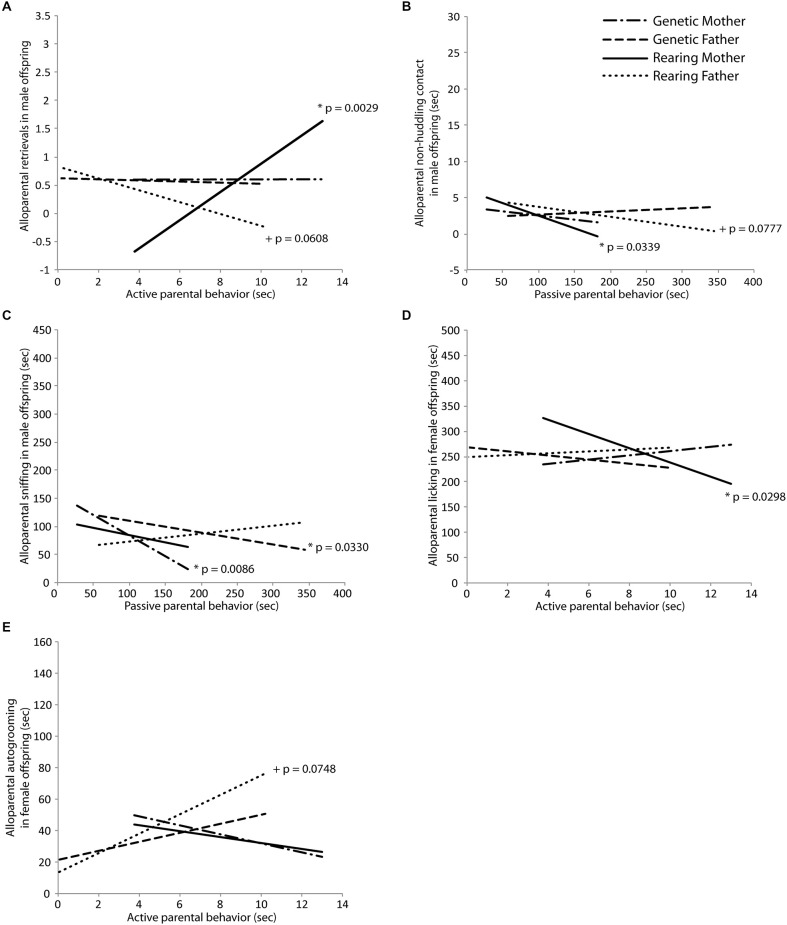
**Intergenerational transmission of parental behavior. (A)** Retrieval in an alloparental care test by male offspring was predicted by early active care behavior in the rearing mother (*t* = 3.55, *p* = 0.0029) and tended to be predicted by the rearing father (*t* = −2.03, *p* = 0.0608). **(B)** Non-huddling contact with an infant by male offspring was predicted by early passive care behavior in the rearing mother (*t* = −2.56, *p* = 0.0339) and tended to be predicted by the rearing father (*t* = −2.02, *p* = 0.0777). **(C)** Sniffing of an infant by male offspring was predicted by early passive care behavior in the genetic mother (*t* = −3.46, *p* = 0.0086) and the genetic father (*t* = −2.57, *p* = 0.0330). **(D)** Licking of an infant by female offspring was predicted by early active care behavior in the rearing mother (*t* = −2.44, *p* = 0.0298) and **(E)** autogrooming in female offspring tended to be predicted by early active care behavior in the rearing father (*t* = 2.39, *p* = 0.0748).

Behavior in an alloparental care test was transmitted primarily in a non-genomic fashion from the rearing parents to both male and female offspring. In male offspring, active care behavior from the rearing mother significantly positively predicted retrieval behavior in the alloparental care test (*t*_(18)_ = 3.55, *p* = 0.0029; Figure 4A), and active care behavior in the rearing father tended to negatively predict alloparental retrievals (*t*_(18)_ = −2.03, *p* = 0.0608; Figure 4A). Passive care behaviors in the rearing father significantly negatively predicted alloparental non-huddling contact (*t*_(18)_ = −2.56, *p* = 0.0339; Figure 4B) while this same behavior in the rearing mother tended to also negatively predict alloparental non-huddling contact (*t*_(18)_ = −2.02, *p* = 0.0777; Figure 4B) for male offspring. Passive care behavior in the genetic mother significantly negatively predicted sniffing behavior in male offspring (mother: *t*_(18)_ = −3.46, *p* = 0.0086; Figure 4C). In female offspring, active care in the rearing mother significantly negatively predicted alloparental licking (*t*_(16)_ = −2.44, *p* = 0.0298; Figure 4D), while active care behavior in the rearing father tended to positively predict autogrooming by the offspring in the alloparental care test (*t*_(16)_ = 2.39, *p* = 0.0748; Figure 4E). Results of Fisher’s combined probability test indicate that the calculated *X*^2^ test statistic is greater than the expected test statistic (50.88926 < 41.94022), suggesting that alloparental behavior of offspring is predicted by the parental behavior of their rearing parent.

## Discussion

Research in rat models of variation in early maternal licking/grooming behavior show that changes in behavioral and neurochemical outcomes in offspring are largely due to environmental factors (Caldji et al., 1998; Francis et al., 1999, 2002; Champagne et al., 2003). We have previously shown that variation in biparental care in the prairie vole is related to changes in behavior and physiology in offspring (Perkeybile et al., 2013; Perkeybile and Bales, 2015) but data presented here suggest that outcomes for different systems may arise from different sources; in this case behavioral outcomes are susceptible to environmental influences while neuroendocrine outcomes appear potentially susceptible to genetic influences. As with several examples of early manipulation in this model species, outcomes here were sex dependent.

Previous work in semi-naturalistic field studies in the prairie vole have demonstrated that parents will readily care for any newborn infant and will not discriminate between their own offspring and unrelated offspring in the nest (Hayes and Solomon, 2004). Indeed, following cross-fostering of offspring on PND 1 we saw no differences in the amount of total care directed toward offspring between sexes or between cross-fostered and in-fostered conditions. This supports the idea that prairie vole parents, in the peripartum period, do not discriminate in care directed to their own and unrelated offspring. As a marker of development, offspring weights were collected on PND 7, following completion of parental care observations. There were no differences in weights at this time point between sexes or fostering conditions, further supporting the notion of equal care toward all offspring in the nest up to this point in development.

Increased passive paternal care received in the first week of life was predictive of increased sniffing of the infant in female offspring as well as increased retrieval of the infant by male offspring. This same passive early paternal care was also associated with a decrease in V1aR binding in the BNST of male offspring, a region implicated in parental behavior (Bester-Meredith et al., 1999; Frazier et al., 2006). However, no relationship was subsequently seen between V1aR binding in male offspring and their behavior in an alloparental care test, indicating that V1aR density in regions involved in displays of parental behavior may not be crucial to displays of alloparenting in male prairie voles. In fact, a previous pharmacological study from our lab found that alloparental care in males could be facilitated through either OTR or V1aR (Bales et al., 2004). While most research on consequences of the early care environment have focused on maternal behavior, very likely due to the rarity of paternal care of offspring in mammals, studies in biparental species are becoming more prevalent and are showing that paternal care, much like maternal care, can influence offspring outcomes. Much of this work has been done in the California mouse, where paternal care has been shown to impact offspring behavior (Marler et al., 2003; Frazier et al., 2006), cognition (Bredy et al., 2004), and neuroendocrine function (Bester-Meredith and Marler, 2003; Frazier et al., 2006). Some work has also been done in the biparental prairie vole, showing that the absence of the father from the natal nest disrupts the development of species-typical alloparental care and pair bonding (Ahern and Young, 2009). Our lab has previously found that fathers in low-contact breeder pairs spend a greater amount of time caring for offspring than do fathers in high-contact pairs and this is correlated with increased social behavior in the offspring (Perkeybile et al., 2013). The present results are consistent with the previous finding in high- and low-contact early care in that with increased early passive paternal care we found, in both cases, decreases in active interaction with the pups, including retrievals and sniffing. We did not, however, see an increase in quiescent contact with the pup in this study as we have previously seen.

No relationship was observed between early maternal care received and later behavior in an alloparental care test, but increased amounts of active maternal care correlated with a decrease in V1aR binding in the CeAmy in male offspring and an increase in OTR binding in the BNST in female offspring. There was also no clear relationship between alloparental behavior and receptor binding in adolescent offspring. This is somewhat surprising given previous research showing that increases in alloparental behavior in females are associated with increased OTR density in the NAcc and caudate putamen as well as decreased OTR density in the LS (Olazábal and Young, 2006a, b). Juvenile prairie voles, both males and females, are typically alloparental (Solomon, 1991; Wang and Novak, 1994). However, there is considerable variation in the reported proportion of naïve adult females that display alloparental vs. infanticidal behaviors (Roberts et al., 1998a; Lonstein and De Vries, 2000, 2001; Olazábal and Young, 2005) and it is likely that variation in OTR density in these regions is responsible for this variation in adult responsiveness toward infants. The present results show no relationship between alloparental behavior and increased early life active maternal care or alloparental behavior and OTR binding. Because this early active maternal care is associated with increased OTR density in the BNST, a region that is important for maternal responsiveness in postpartum females (Insel, 1992; Meddle et al., 2007; Perrin et al., 2007), this would suggest that regulation of alloparenting in naïve animals and maternal behavior of own offspring may be regulated by different neural circuits.

The influence of early parental behavior on receptor binding in the OT and AVP systems in the current study appears to be sexually dimorphic, with changes in OTR seen in female offspring and changes in V1aR in male offspring. This is consistent with the idea that, while both neuropeptides are present in both sexes, the OT system may be more active in females and the AVP system more active in males. This differential function provides a potential mechanism for the sexually dimorphic outcomes often seen following early manipulations. For example, in the prairie vole early exposure to OT alters several behaviors as well as both the OT and AVP systems (Carter, 2003; Carter et al., 2009) while early handling manipulations also alter behavior and neurochemistry in offspring (Bales et al., 2007a, 2011). In addition, several social behaviors are modulated differently between sexes, including alloparental behavior (Bales et al., 2004; Olazábal and Young, 2006a, b; Kenkel et al., 2012), pair bonding (Winslow et al., 1993; Williams et al., 1994), and parental behavior (Insel and Shapiro, 1992b; Bamshad et al., 1993, 1994). Sex-dependent changes in neuropeptide receptor systems following early biparental care here supports this notion of differential importance and actions of OT and AVP in males and females and fits with data in the rat, where varying early maternal licking and grooming impacts OTR density in the female offspring and V1aR density in the male offspring (Francis et al., 2002).

From the perspective of the offspring, social interactions in the post-natal environment come almost exclusively from parents and littermates. There is considerable evidence that this early natal environment has the potential to shape offspring development and, in particular, that parental characteristics are transmittable to offspring. In rats, maternal licking and grooming behavior is transmitted in a non-genomic fashion to female offspring (Francis et al., 1999). In the biparental California mouse, a species in which paternal involvement is vital for offspring survival, decreases in paternal care of male offspring lead to similar decreases in infant care in the subsequent generation (Bester-Meredith and Marler, 2003; Gleason and Marler, 2013), indicating that paternal behavior can be non-genomically transmitted similarly to maternal behavior. Beyond rodent models, there is evidence that behavior of the mother can alter behavior displayed by offspring in macaques (McCormack et al., 2006; Maestripieri et al., 2007), vervet monkeys (Fairbanks, 1989), zebra finches (Naguib et al., 2006), and Japanese quail (Formanek et al., 2008; Pittet et al., 2013). Our results show several trends that suggest receptor binding patterns tend to be transmitted from parent to offspring in a genomic fashion, with sex-dependent effects of the genetic mother and father on regions associated with parental behavior, including the CeAmy, BNST, LS, and NAcc core. In all cases, these trend associations between parent and offspring receptor binding were sex-specific, with binding in genetic fathers predicting male offspring binding and binding in genetic mothers predicting female offspring binding, with the exception of the NAcc core tending to be predicted by binding of both the genetic mother and father for male offspring. Behavioral transmission, however, appeared to follow a non-genomic transmission pattern—in both male and female offspring, alloparental behavior tended to be predicted by the behavior of the rearing parents, with the exception of sniffing behaviors in male offspring. This follows findings in several other species previously discussed of non-genomic transmission of maternal and paternal behavioral phenotypes.

Maternal behavior in rat dams can be classified as active or passive behaviors. Terkel et al. (1979) termed active behavior as motivated maternal behavior because dams must initiate the behavior, typically with physical movement. These behaviors may include licking and grooming of pups, nest building, and retrieval and have been referred to as pronurturant behaviors because they often promote nursing (Stern, 1996). In contrast, nursing postures were classified as passive behavior in that they can be initiated by offspring and require only passive participation by the dam (Terkel et al., 1979). Much of the work investigating non-genomic transmission of traits to offspring has focused on the impact of active behaviors, including the consequences of high compared to low amounts of licking and grooming on adult offspring maternal behavior in rats (Francis et al., 1999; Champagne et al., 2003) as well as the effects of impaired maternal care received on retrieval behavior in female mice (Curley et al., 2008). The impact of passive care on non-genomic transmission of traits, however, is considerably less studied.

Here we demonstrated that active maternal care predicted increases in active alloparental behavior in male offspring but decreases in active alloparental behavior in female offspring. Meanwhile, passive care from the rearing mother predicted decreases in later passive alloparental behavior only for male offspring. This indicates that early active maternal care impacts the display of similar active behaviors in adolescence and early passive care does the same for expression of later passive care behaviors, suggesting a somewhat linear non-genomic transmission of behavioral characteristics. There were, however, only trends for paternal care to predict decreases in both active and passive pup-directed behaviors in male but not female offspring. Similar to maternal behavior, active paternal care tended to predict active alloparental behavior while passive paternal care tended to predict passive alloparental behavior. The trends in paternal care impacts suggests that, while the rearing father does play a role in shaping male offspring behavior, the rearing mother has a greater overall influence. This is perhaps not surprising given the amount of time offspring spend with the mother compared to the father early in life—pups spend a much greater amount of time with the mother during this time, primarily due to the need to nurse.

The lack of effect of the rearing environment on receptor binding suggests a strong genetic influence on receptor binding; however, this is not necessarily consistent with other studies of early developmental influences in prairie voles (Bales et al., 2007b, 2011). One possible explanation is that in this particular sample, there were relatively low levels of variation in the care directed toward offspring in the first postnatal week. There were less than 400 s of difference between the highest and lowest amount of total parental pup-directed care observed in the first week. We have previously observed a range of nearly 1300 s in pup-direct care in the first days following birth (Perkeybile et al., 2013) in a larger set of breeder pairs, suggesting that the pairs involved in this study were more similar to one another in their behavior toward offspring. If parents in this experiment do not exhibit the widest range possible in behavior and receptor distribution, one possibility is that the early care environment offered by the genetic parents does not differ greatly from that of the rearing parents and, hence, offspring experienced similar care from their rearing parents as they would from their genetic parents. Alternatively, there is a great deal of intraspecific variation in receptor binding density in this species. For example, variation in OTR densities have been linked to variation in displays of alloparental care (Olazábal and Young, 2006b) and increasing OTR expression during development leads to enhanced alloparental behavior (Keebaugh and Young, 2011). It may be that variations seen here in offspring binding densities are primarily due to individual variation, although this may still be passed to offspring via genomic mechanisms. Another possibility is that our current understanding of the brain areas and receptor types involved in alloparental care may still need further study.

In summary, we present evidence for the potential for differential modulation of behavior and neuroendocrine receptor distribution in offspring following cross-fostering, where inheritance of behavioral phenotypes occurs via non-genomic mechanisms while inheritance of OTR and V1aR density trends toward happening through genetic mechanisms. In all cases, relationships between parent and offspring were sex-dependent, adding to the literature on sexually dimorphic responses to early life experiences. Future work should focus on investigating the intergenerational transmission of a broader array of behavior and work toward establishing a causal link between early life factors, such as parental behavior and physiology as well as environmental conditions, and outcomes in offspring. The findings presented here suggest that behavior of male and female offspring is differentially influenced by their rearing environment while their OTR and V1aR systems show trends toward being influenced primarily by their same-sex genetic parent.

## Conflict of Interest Statement

The authors declare that the research was conducted in the absence of any commercial or financial relationships that could be construed as a potential conflict of interest.

## Footnotes

^1^www.behaviortracker.com
